# Dual malignancies: a case report of the sequential occurrence of a trichilemmal tumor and breast carcinoma in a 56-year-old female

**DOI:** 10.3389/fonc.2025.1590725

**Published:** 2025-11-26

**Authors:** Bhargav Vuppumalla, A. Ravichandran, M. Manickavasagam, Chaitanya Kumar Javvaji, Keta Vagha

**Affiliations:** 1 Department of Medical Oncology, Sri Ramachandra Medical College and Research Institute, Sri Ramachandra Institute of Higher Education and Research, Chennai, India; 2 Department of Medical Oncology, Jawaharlal Nehru Medical College, Datta Meghe Institute of Higher Education and Research, Wardha, India; 3 Department of Pediatrics, Jawaharlal Nehru Medical College, Datta Meghe Institute of Higher Education and Research, Wardha, India

**Keywords:** trichilemmal tumour, breast carcinoma, chemotherapy, ductal carcinoma, targeted therapy, discordant response

## Abstract

The authors present a rare case of the sequential occurrence of a malignant trichilemmal tumor (MPTT) and invasive ductal carcinoma of the breast in a 56-year-old woman. The patient initially presented with a palpable breast lump and a longstanding asymptomatic scalp swelling that had been present since childhood. Histopathological evaluation confirmed the breast lump as invasive ductal carcinoma, while the scalp swelling, initially suspected to be a benign cyst, was diagnosed as an MPTT. The patient underwent combination chemotherapy, which resulted in a favorable response of the breast carcinoma; however, the MPTT exhibited a discordant therapeutic outcome. Trichilemmal carcinoma is an uncommon cutaneous neoplasm, and its coexistence with breast carcinoma represents an exceedingly rare clinical scenario. Furthermore, the differential response of these malignancies to chemotherapy presents a significant therapeutic challenge. This case underscores the importance of accurate diagnosis, individualized treatment strategies, and a multidisciplinary approach for optimally managing such complex oncological presentations.

## Introduction

Malignant proliferating trichilemmal tumor (MPTT) is an extremely rare adnexal tumor in the malignant spectrum of proliferating pilar tumors. Histologically, it is characterized by excessive proliferation of the outer sheath epithelium of the hair follicles (1). Proliferating trichilemmal tumors (PTT) comprise 0.1% of all skin tumors. Additionally, most patients who present these lesions are older women and. in 90% of the cases, these tumors occur on the scalp (1). The pathophysiology of trichilemmal carcinoma is still unclear. Radiation from the sun is one factor that causes the lesions’ growth due to its location and distribution (2). PTTs rarely exhibit malignant transformation (3). Trichilemmal cysts can also transform into malignant trichilemmal carcinomas due to p53 deletion.

Breast cancer (BC) is the most common malignant tumor in women and one of the principal causes of cancer mortality in this sex, despite there being significant improvements made in treatments in recent decades. HER2-positive breast cancer is an aggressive subtype that accounts for 15% to 20% of invasive breast cancers. HER2-positive breast cancers tend to grow and spread faster than other types of breast cancer (4). However, over the last years, several therapeutic advances have improved the clinical treatment of HER2+ disease and, thus, its prognosis.

Breast cancer, being a very heterogenous disease, requires timely clinical decisions made with clinical acumen and a comprehensive insight into the tumor’s molecular profile to predict the disease’s probable clinical outcome. The concomitant or sequential occurrences of this with other tumors make the treatment more challenging. There is very limited data for these two very unique tumors occurring in an individual, hence we report our experience from the department of Medical Oncology in Sri Ramachandra Institute of Higher Education and Research, Chennai, India, with a case of trichilemmal carcinoma presenting sequentially with right breast mass and regional axillary lymph nodal metastasis treated with chemotherapy and wide local excision.

## Case presentation

A 56-year-old Indian woman presented with a recent painless palpable lump in her right breast, which she noticed incidentally while taking a shower one month previously. She also reported a scalp swelling on the left side of the nape of her neck that had existed since childhood, initially appearing around her high school years around 14 years of age as a peanut size, painless swelling well concealed in her dense hair. The patient never got the swelling evaluated. The patient also revealed that the scalp swelling had recently increased in size over the previous 3 months. The patient had no associated comorbidities or any known familial cancers. There were no relevant past interventions. On examination of her right breast, a single non-tender lump of size 3 x 3cm in the upper outer quadrant was revealed; the lump was firm to hard in consistency with Peau d’orange skin changes and a 1x1cm solitary firm axillary lymph node. Local examination of the scalp showed a single swelling of size 6x5 cm present over the left side of the nape of the neck and the scalp, firm to hard in consistency.

A mammography revealed a BIRADS 5 lesion correlating with USG, an irregular taller than wider hypoechoic lesion measuring 1.6x1.4x1.2 cm (AP*TR*CC) with spiculated margins. The lesion was 6.1 cm away from the nipple and 0.7 cm deep to the skin surface. Color Doppler revealed increased vascularity. The overlying skin appeared mildly thickened, measuring 4 mm in thickness. The right axilla showed a few enlarged lymph nodes with asymmetrical cortical thickening, the largest measuring 1.8 x 1.2 cm.

**Figure 1:**
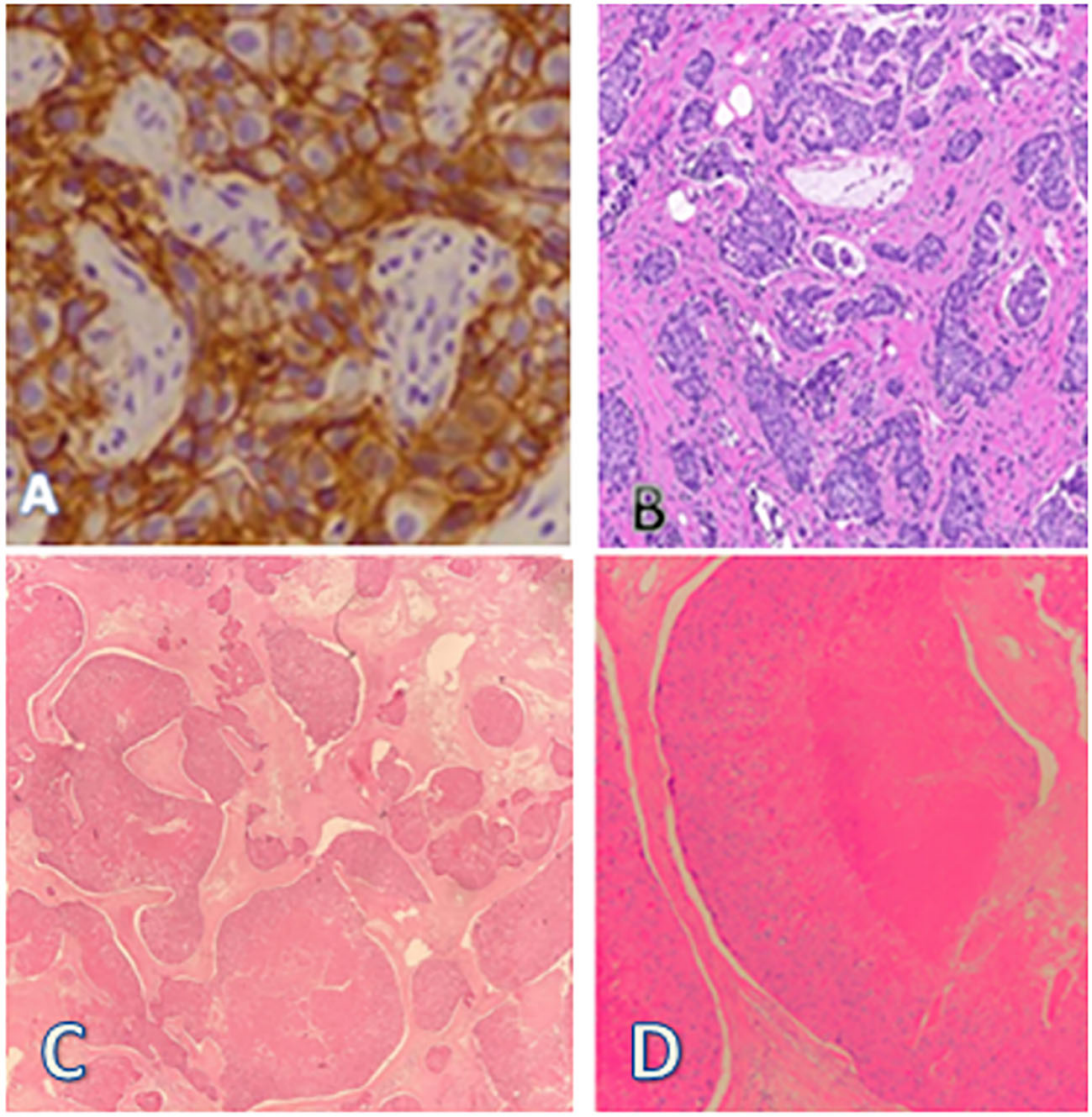
**(A)** Her2neu-enriched molecular subtype of **(B)** invasive mammary carcinoma **(C)** Linear cores of tissue showing lobules of squamous proliferation with trichilemmal differentiation showing moderate nuclear atypia, occasional mitosis, and necrosis. **(D)** Areas of dense laminated eosinophilic keratinization noted.

A USG-guided core needle biopsy of the right breast lesion revealed invasive mammary carcinoma, grade 2, and immunohistochemistry pattern favoring the Her2-enriched molecular subtype. The lesion was ER negative, PR negative and Ki67 80%. (**Figures 1A, B**).

A USG-guided biopsy was taken the very next day from the scalp swelling and showed a malignant trichilemmal tumor, with the immunohistochemistry pattern favoring the diagnosis with loss of CD34 (**Figures 1C, D**).

**Figure 2:**
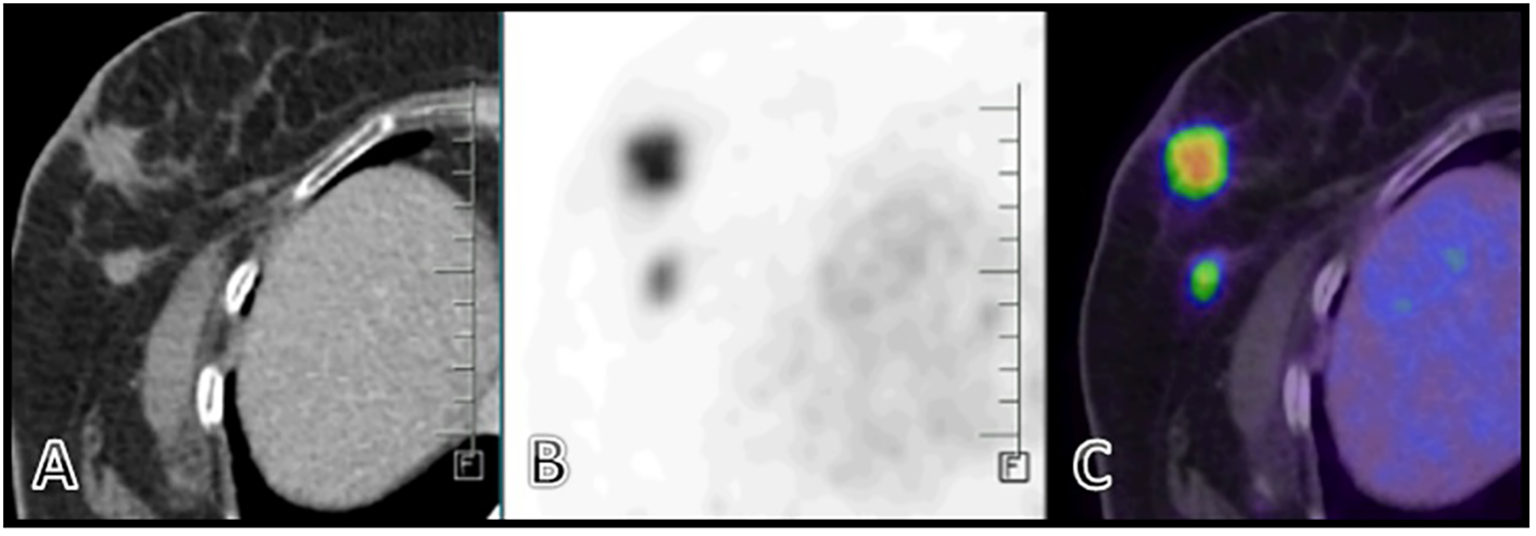
**(A–C)** Ill-defined spiculated FDG avid (SUV max 10.8) mass lesion seen in the outer quadrant of the right breast measuring 22 x 21 x 21 mm,. A few thin fibrous strands seen extending from the lesion to the overlying mildly thickened skin (2.5mm). Two other FDG avid nodules were also seen.

**Figure 3:**
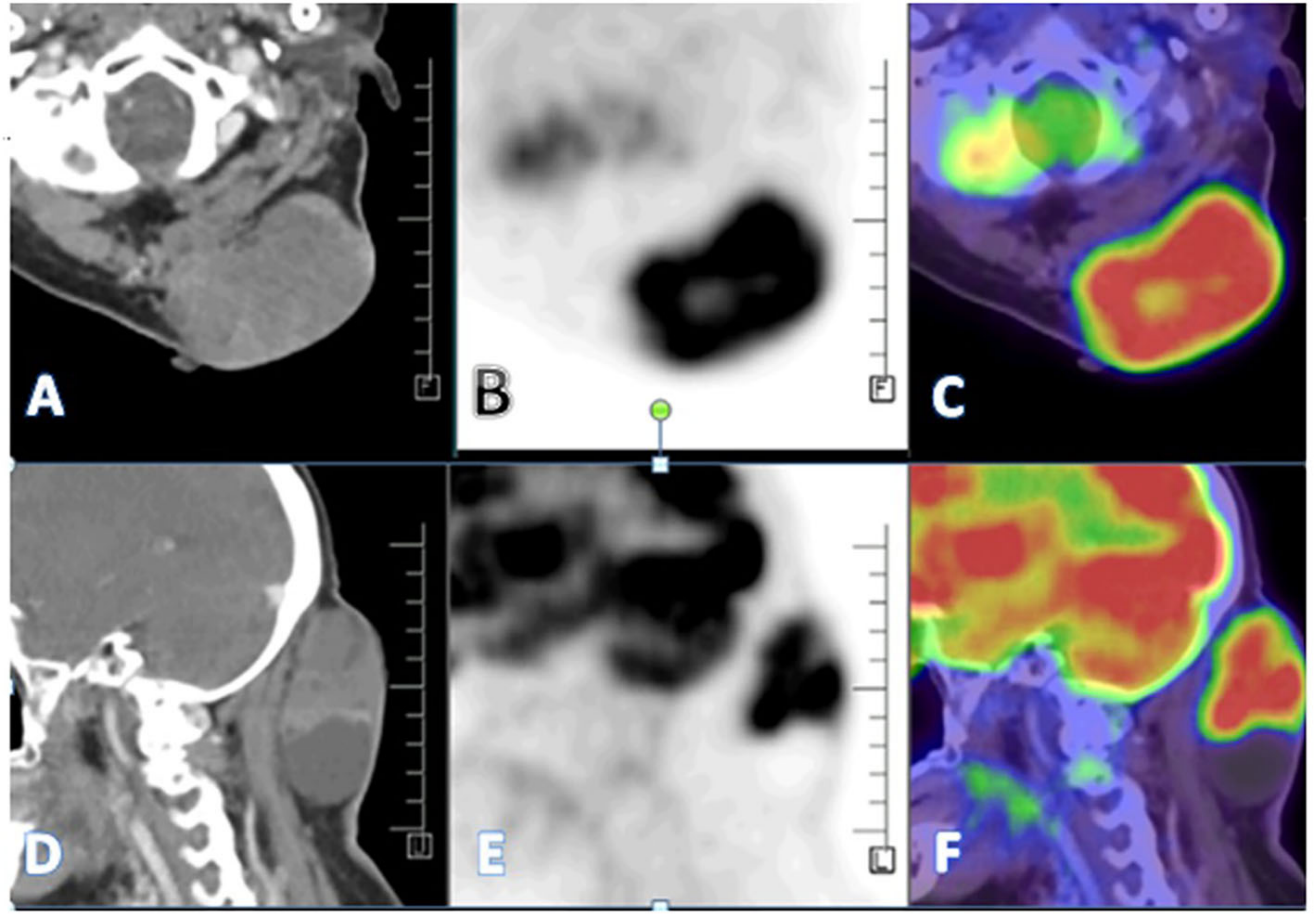
**(A–F)** - A large heterogeneously enhancing FDG avid (SUV max 11.8) solid cystic lesion seen in the left paramedian aspect of the nape of the neck measuring 67 x 41 x 75 mm involving overlying skin and closely abutting the underlying muscles-likely malignant.

A PET CT scan showed multiple FDG-avid, malignant-looking lesions in the outer quadrant of the right breast (**Figures 2A–C**); right level I, II, and III axillary lymphadenopathy; and a large, malignant-looking solid cystic lesion on the left side of the nape of the neck, involving the scalp and closely abutting the underlying muscles (**Figures 3A–F**). With all the above data, the patient was diagnosed with a HER2-positive Invasive mammary carcinoma (mcT4bN1M0) and Malignant proliferating trichilemmal tumor.

After consolidating the findings, it appeared that the occurrences were sequential. The tumor likely had an indolent course over several years, remaining unevaluated until its recent increase in size. In contrast, the breast lesion seemed to be of more recent onset, probably developing over a few months, given its aggressive histology and non-metastatic presentation. This favored the interpretation of a sequential occurrence, despite both being diagnosed simultaneously.

The definitive first-line treatment for Trichilemmal tumors is, ideally, surgical excision with or without Mohs micrographic technique; however, in view of Her2-positive breast cancer being an aggressive tumor, a multidisciplinary tumor board discussion was held regarding the optimal management strategy. The panel weighed the benefit of upfront surgery for the trichilemmal tumor as the ideal option against neoadjuvant therapy for breast cancer given its aggressive histological subtype and the potential risk of disease progression if scalp surgery caused delays. A third option considered was the simultaneous surgical removal of both tumors, followed by adjuvant chemotherapy; however, this approach would forfeit the advantages of neoadjuvant therapy, including the opportunity to assess tumor response and gather valuable information to guide further treatment. The option of upfront surgery to the scalp tumor and then neoadjuvant treatment for the breast imposed the risk of delaying breast cancer treatment due to potential surgical complications. Ultimately, the board concurred that neoadjuvant chemotherapy had a therapeutic importance over the trichilemmal tumor surgery, which otherwise was asymptomatic for years, and should be initiated. Hence, the treatment decided upon was eight cycles of NeoAdjuvant chemotherapy followed by surgery for breast carcinoma and trichillemal tumor and subsequent adjuvant trastuzumab therapy. Adriamycin, being a common chemotherapeutic drug for both the tumors neoadjuvant chemotherapy, was given and AC-TH protocol was followed. Dual Her2 blockade was not considered due to financial constraints. The planned line of treatment was to provide neo- adjuvant chemotherapy with four cycles of doxorubicin (60mg/m2) and cyclophosphamide(600mg/m2) followed by four cycles of docetaxel (75mg/m2) and trastuzumab (loading 8mg/kg followed by 6mg/kg) given as 21 day cycles, constituting a total of eight neoadjuvant chemotherapy cycles followed by modified radical mastectomy of the right breast and wide local excision of the trichilemmal tumor, after which the patient would be reassessed and adjuvant therapy with Herceptin would be continued for a year.

After three cycles of chemotherapy, clinical reassessment revealed a significant reduction in the size of the breast lump and axillary lymph nodes. However, by the third cycle, the patient developed an increase in the size of the scalp lesion (8 × 7 cm), which was associated with pain and was interfering with her daily activities After a multidisciplinary tumor board discussion, the board concurred to perform a wide local excision of the scalp lesion, given acute pain at the site and lack of therapeutic response, and to complete the remaining five cycles of the initial neoadjuvant chemotherapy regimen as per the treatment protocol. The patient underwent wide local excision of the trichilemmal tumor. Three weeks after the excision, the patient continued on the initially planned chemotherapy schedule. Following the completion of neoadjuvant chemotherapy, the patient underwent a modified radical mastectomy (MRM). Histopathology revealed a complete pathological response. Subsequently, post-surgery, the patient received one year of maintenance trastuzumab therapy.

The patient is currently being followed up off treatment, is asymptomatic, and clinically does not show any signs of carcinoma relapse.

A summarizing sequence of events with relevant data of this case is explained with a listing in **Table 1**.

**Table 1:** Sequential events of the case.

Sequential events of the case	Interpretation with relevant data
Case presentation (Symptoms and history)	A 56-year-old woman came with a palpable right breast lump for one month. She also had scalp swelling since childhood (which was not addressed previously) that had gradually increased in size over the last 3 months.
Clinical Examination	Right breast: A single non-tender lump of size 3 x 3cm felt in the upper outer quadrant of the right breast, firm to hard in consistency, Peau d’orange appearance seen. The scalp showed a single swelling of size 6 x 5 cm present over the posterior aspect of the scalp, which was firm to hard in consistency
Investigations	USG-guided biopsy from scalp swelling malignant trichilemmal tumor with loss of CD34 USG-guided Tru-cut biopsy of right breast IMC Her2-enriched molecular A PET CT scan showed multiple malignant-looking lesions in the right breast; right level I, II, and III axillary lymphadenopathy; and a large, malignant-looking solid cystic lesion involving the scalp on the left side of the nape of the neck.
Diagnosis	HER2-positive Invasive mammary carcinoma (mcT4bN1M0) and Malignant proliferating trichilemmal tumor
Treatment	Three cycles of Neo Adjuvant chemotherapy followed by wide local excision (done due to discordant response of tumor) followed by five cycles of chemotherapy followed by MRM. Adjuvant Trastuzumab was given for a year.
Follow up	Asymptomatic, clinically does not show any signs of relapse
Timeline summary	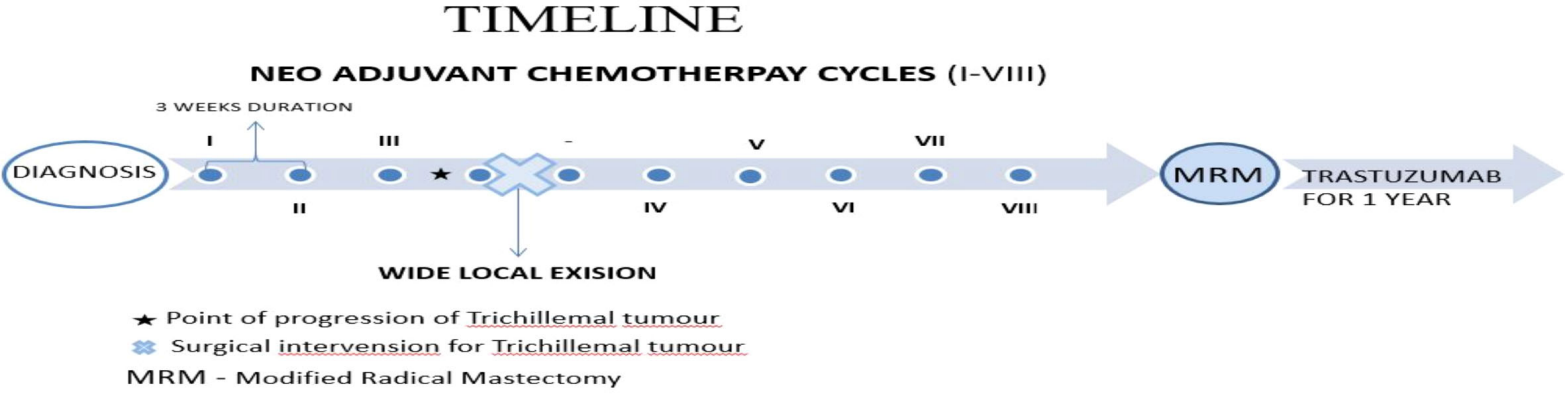

## Patient’s perspective

From my point of view, this cancer journey was complex and brought significant physical, emotional, and financial challenges. Before my diagnosis, I realized there was a noticeable gap between how much I thought I understood about the disease and the actual knowledge I needed to cope with it. During chemotherapy, the most difficult side effects I faced were alopecia, nausea, fatigue, and loss of appetite. Despite these struggles, I felt reassured by the coordinated efforts of the hospital team. The multidisciplinary board discussions and the holistic approach of my doctors gave me confidence and helped me feel supported throughout the treatment process.

## Discussion

Breast carcinoma and trichilemmal tumors, particularly trichilemmal carcinoma (TC), represent a rare clinical scenario where both malignancies occur simultaneously in a patient. Trichilemmal carcinoma is a malignant neoplasm derived from the outer root sheath of hair follicles, typically presenting as an indolent skin lesion. However, it can exhibit aggressive behavior, including local invasion and metastasis, particularly in cases with a history of other malignancies, such as breast cancer (5). Trichilemmal cysts can also transform into malignant trichilemmal carcinomas due to p53 deletion. The clinical features of malignant transformation result from the combination of an overlying area of scalp alopecia, with a size >5 cm, locations other than the scalp, accelerated growth, and areas of ulceration (6). Histologically, one observes increased mitotic activity and increased proliferative index and activity beyond the basal layer of the skin. Other features include characteristic trabeculae with peripheral palisading of cells extending to the level of the dermis (6).

TC has a non-aggressive course (7), despite its aggressive histology. The prognosis is generally good, as it has low metastatic potential, similar to cutaneous squamous-cell carcinoma (SCC).

HER2-positive breast cancer is characterized by aggressive biological behavior and poor prognosis. The advent of anti-HER2 targeted therapies has significantly improved patient outcomes (8). HER2, a transmembrane glycoprotein with a molecular weight of 185 kDa, comprises extracellular, transmembrane, and intracellular domains that serve as the target for anti-HER2 therapies (8).

Recent literature highlights instances where patients with breast cancer have developed TC, either as a metachronous or synchronous tumor. For example, one study documented a case of metastatic TC in a patient with a history of breast cancer, emphasizing the importance of careful monitoring for secondary malignancies in breast cancer survivors (6).

The co-occurrence of these tumors necessitates a multidisciplinary approach to diagnosis and management, considering the potential for aggressive disease progression and the implications for treatment strategies.

Treatment options for HER2-positive breast cancer are currently based on the CLEOPATRA trial, which established the combination of pertuzumab, trastuzumab, and docetaxel as the standard of care for metastatic HER2-positive breast cancer (9). However, since the tumor board recommended including Adriamycin due to its potential activity against the trichilemmal tumor, the treatment protocol adopted was the AC-TH regimen. Although Adriamycin is associated with significant toxicity, it remains highly effective in HER2-positive breast cancer (9).

The landmark adjuvant trastuzumab trials by Slamon et al. and others demonstrated that adding trastuzumab to standard chemotherapy—often consisting of anthracyclines such as Adriamycin plus cyclophosphamide followed by taxanes—significantly improved disease-free and overall survival. These findings established trastuzumab as a standard component of adjuvant therapy for HER2-positive breast cancer. Due to financial constraints, dual HER2 blockade was not administered in this case (10).

Treatment options for metastatic trichilemmal carcinoma (TC) remain limited due to the rarity of the disease and the absence of standardized protocols. However, several approaches are generally considered. Wide excision with clear margins, typically around 1 cm, serves as the primary treatment for localized tumors. This approach is crucial for curative intent, particularly in cases without metastasis (11). Mohs micrographic surgery is another preferred technique, especially in cosmetically sensitive areas, as it facilitates preserving surrounding healthy tissue while ensuring complete removal of malignant cells (12). Surgery margin and lymph nodes metastasis were prognostic factors that influenced the treatment outcome (2).

Systemic chemotherapy may be considered for patients with distant metastases, although no specific regimen has been established for trichilemmal carcinoma. Chemotherapeutic agents commonly used for advanced squamous cell carcinoma, such as cisplatin, doxorubicin, and cyclophosphamide, have been utilized in some cases (13). In select instances, topical treatments, including imiquimod (5% cream), have demonstrated effectiveness. Cisplatin in combination with either Adriamycin, cyclophosphamide, 5-FU, or vindesine and bleomycin have slowed the progression of metastatic disease but has not been curative (13–15). However, its use remains controversial due to concerns about masking recurrence and should only be considered when surgical intervention is not a viable option (16). A tumor that progressed after multiple surgeries, radiation, and chemotherapy, has achieved good response and disease control with pembrolizumab, an immune checkpoint inhibitor targeting programmed cell death protein-1 (17).

In summary, this case report presents a rare instance of coexisting malignant proliferating trichilemmal tumor (MPTT) and invasive ductal carcinoma of the breast in a 56-year-old woman. The patient initially presented with a palpable breast lump and an asymptomatic scalp swelling, the latter of which had been present since childhood. Histopathological evaluation confirmed the diagnosis of HER2-positive invasive ductal carcinoma, while the scalp lesion, initially suspected to be a benign cyst, was identified as an MPTT. Neoadjuvant chemotherapy was administered for the breast carcinoma, resulting in significant tumor regression; however, the MPTT demonstrated a discordant response, raising therapeutic challenges. The patient subsequently underwent wide local excision of the scalp lesion and a modified radical mastectomy after completion of neoadjuvant chemotherapy, followed by trastuzumab therapy. Post-treatment follow up revealed no evidence of recurrence.

While HER2-positive breast carcinoma typically responds well to targeted therapy and systemic chemotherapy, trichilemmal carcinoma had no response to systemic chemotherapy. However, its treatment approach lacks standardized treatment guidelines, making management particularly challenging. The discordant response observed in this case underscores the need for individualized treatment strategies. It reinforces the role of histopathological examination in accurately diagnosing seemingly benign lesions, emphasizing the importance of timely diagnosis and a tailored multidisciplinary treatment approach.

## Conclusion

This case underscores the diagnostic and therapeutic challenges associated with managing multiple malignancies with differing biological behaviors. The discordant response of these tumors to neoadjuvant chemotherapy emphasizes the need for individualized treatment strategies and a multidisciplinary approach in oncology, involving surgical, medical, and pathological expertise to optimize patient outcomes. Early histopathological evaluation played a crucial role in identifying the scalp lesion as malignant, preventing potential delays in management. Given the rarity of malignant trichilemmal tumors and the lack of standardized treatment guidelines, this case adds to the limited literature with valuable insights into their clinical course and response to therapy. It reinforces the importance of further research to establish optimal therapeutic protocols.

Future studies are needed to further investigate the molecular mechanisms underlying such rare coexisting malignancies, to establish evidence-based treatment protocols, and to follow up cases of MPTT for potential risks of developing other malignancies, such as breast cancer, and vice versa. Ongoing surveillance and long-term follow up are essential to monitor recurrence and improve patient outcomes in such complex oncological scenarios.

## Data Availability

The original contributions presented in the study are included in the article/supplementary material. Further inquiries can be directed to the corresponding author.
